# Cefepime resistance with preserved ceftriaxone susceptibility in *Proteus mirabilis* osteomyelitis associated with *bla*_OXA-1_ gene amplification: Two case reports with genomic analysis

**DOI:** 10.1016/j.idcr.2026.e02654

**Published:** 2026-06-25

**Authors:** Amorce Lima, Mackenzie Collins, Mario Jaramillo, Shekina Gonzalez-Ferrer, Anita Sircar, Paul R. Allyn, Shangxin Yang

**Affiliations:** a Department of Pathology and Laboratory Medicine, David Geffen School of Medicine, University of California, Los Angeles, CA, USA; b Division of Infectious Diseases, David Geffen School of Medicine at UCLA, Los Angeles, CA, USA

**Keywords:** Proteus mirabilis, Osteomyelitis, Cefepime resistance, Gene duplication, OXA-1

## Abstract

**Objective:**

Retrospective case series to describe an unusual cefepime-resistant, ceftriaxone-susceptible phenotype in *Proteus mirabilis* osteomyelitis associated with *bla*_OXA-1_ gene amplification.

**Patients:**

Two adult patients with diabetes mellitus and chronic osteomyelitis, one with diabetic foot ulcer and one with infected hip prosthesis, both with prolonged antibiotic treatment.

**Methods:**

Clinical data and antimicrobial susceptibility results were reviewed. Whole-genome sequencing was performed using short-read and long-read platforms with hybrid assembly. Resistance gene identification, phylogenetic analysis, and gene copy number estimation were conducted.

**Results:**

Three isolates of *P. mirabilis* from two patients demonstrated an unusual phenotype of cefepime resistance (MIC 16–32 μg/mL) with susceptibility to ceftriaxone. Hybrid assembly and coverage analysis demonstrated amplification of the *bla*_OXA-1_-containing cassette, with approximately 16- and 24-fold duplication in patient 1 and 6-fold duplication in patient 2. Increased copy number correlated with cefepime resistance. All isolates harbored the multidrug resistance cassette containing *bla*_OXA-1_ within an IS6-family (IS26-like) transposon, which are found to widely circulate in plasmids or chromosomes of *P. mirabilis* and many other Enterobacterales species.

**Conclusions:**

This study showed that duplication of a transposon-associated *bla*_OXA-1_-containting cassette in *P. mirabilis* can confer cefepime resistance while preserving ceftriaxone susceptibility. In the outpatient setting, recurrent infection during prolonged therapy may select for gene amplification–mediated resistance with atypical susceptibility patterns, complicating antibiotic selection. This highlights the need to monitor resistance and reassess treatment strategies in cases of chronic osteomyelitis that are not improving or worsening on therapy.

## Introduction

Osteomyelitis is a serious inflammatory condition of the bone and its structures. It can occur due to hematogenous seeding of microorganisms from a distant source, extension into the bone from an adjoining site, or by direct inoculation following trauma or surgery [1]. Acute infection may be associated with a sudden onset of fever, pain, and edema of the affected site in the absence of necrotic tissue until days or weeks after the initial infection [2]. Chronic osteomyelitis develops after several months or years of persistent infection and is characterized by necrosis of bone and fistulous tracts from skin to bone [3]. Diabetic patients are at a high risk of developing osteomyelitis, especially involving the foot as a complication of a chronic foot ulcer or soft-tissue infection and can be challenging to manage [4], [5], [6], [7].

*Staphylococcus aureus* and *Pseudomonas aeruginosa* are the two most common organisms isolated among all types of osteomyelitis [2]. Other bacteria include coagulase-negative *Staphylococci*, *Streptococc*i, *Enterococc*i, Enterobacterales, and anaerobes. Patient population-associated microbiologic patterns exist; for instance, osteomyelitis in pediatric patients is more frequently caused by *S. aureus*, *S. pneumoniae*, *S. agalactiae, Kingella kingae,* and aerobic Gram-negative bacteria, while *P. aeruginosa* and *Candida* species are more common etiologies of osteomyelitis associated with injection drug use [4].

*Proteus mirabilis*, a common cause of uncomplicated urinary tract infections and skin and soft tissue infections, has been reported to cause osteomyelitis [5], [6], [7], [8], [9]. Between 30% and 50% of adult vertebrate osteomyelitis caused by *P. mirabilis* are associated with urinary tract infections; however, predisposing factors for *P. mirabilis* osteomyelitis include weakened immune system, renal failure, chronic wounds, and diabetes mellitus [5], [9]. Diagnosis of osteomyelitis relies on both collection of the appropriate specimen (i.e. bone biopsy, blood) and radiographic imaging modalities such as X-Ray, CT, or MRI. Management typically requires prolonged antibiotic therapy and often surgical intervention or amputation depending on the severity of the patient’s condition. Here, we describe two cases of chronic osteomyelitis caused by *P. mirabilis* demonstrating an unusual phenotype of cefepime resistance with preserved ceftriaxone susceptibility. We further characterize the genomic mechanism using hybrid assembly.

## Methods

Antimicrobial susceptibility testing was performed by broth microdilution manually using a gram-negative panel prepared in house, which provided minimal inhibitory concentrations (MICs) and interpretations following the Clinical and Laboratory Standards Institute (CLSI) guidelines (https://clsi.org/).

For whole-genome sequencing (WGS) analysis, genomic DNA was extracted from the isolates using Qiagen EZ1 tissue kit on the EZ1 Advanced XL instrument (Qiagen). DNA concentrations were measured using Qubit™ 1X dsDNA High Sensitivity Assay (ThermoFisher). Short-read sequencing libraries were generated using the Revelo DNA-Seq Enz kit on MagicPrep™ NGS system (Tecan) and libraries were sequenced on the Illumina MiSeq Sequencing System (Illumina). Long-read sequencing libraries were sequenced on Nanopore MinION (Oxford Nanopore Technologies) R10.4.1 flow cell using SQK-RPB114.24 rapid barcoding protocol. Single nucleotide polymorphism (SNP) analysis was performed using the CLC Genomics Workbench version 22.0.2 (Qiagen) using the following parameters: minimum coverage = 10X; minimum allele frequency = 85%; minimum quality = Q15; reference genome = NZ_CP029725. ResFinder database was used to predict genomic resistance markers. Mutational analysis and copy number calculation were performed using Geneious Primer software v2024.0.2 (Dotmatics).

Hybrid genome assembly was performed using Unicycler (v0.5.1) [10], integrating long- and short-read sequencing data. Assemblies were polished using Polypolish (v0.6.1) [11] with Illumina reads to improve base-level accuracy prior to downstream analyses. Genome annotation was performed using Bakta (v1.12.0) [12], and plasmid reconstruction and mobility characterization were conducted using MOB-suite (v3.1.9) [13].

Annotated assemblies, including insertion sequences and transposases, were examined for adjacent mobile genetic elements to evaluate the genomic context of the OXA β-lactamase gene. Illumina reads were mapped back to the assembled genome using BWA-MEM (v0.7.19) [14], and read depth was calculated using samtools (v1.22.1) [15]. Copy number was estimated by comparing the short-read and long read mean depth of the OXA locus to the average chromosomal coverage in the hybrid assembly.

## Case presentations

Patient 1 is a 49-year-old male with a history of insulin-dependent type 2 diabetes mellitus and end-stage renal disease on hemodialysis. He presented to our hospital with nausea, chills, worsening right hallux swelling, pain, and foul-smelling discharge at the site of an existing diabetic foot wound with necrosis. Relevant admission laboratory values included an elevated white blood cell count of 14.2k/μL, erythrocyte sedimentation rate (ESR) of 35 mm/hr (reference range ≤12 mm/hr), and a C-reactive protein (CRP) of 30.9 mg/dL (reference range <0.8 mg/dL). Magnetic Resonance Imaging (MRI) of the right foot showed osteomyelitis of the first distal phalanx with faint marrow edema along the proximal portion. He was empirically started on cefepime, metronidazole, and vancomycin and subsequently underwent debridement of the necrotic tissue. An intra-operative bone culture grew ceftriaxone-susceptible *P. mirabilis* (MIC < 1 mcg/mL) that was also susceptible to cefepime (MIC < 0.5 mcg/mL) (Table 1; Isolate 1). His regimen was adjusted to cefepime monotherapy for ease of dosing post-hemodialysis, and he was discharged with a planned 6-week course.

**Table 1:** Antibiotic susceptibility results of *Proteus mirabilis* isolates.

**Drug**	**Isolate 1**		**Isolate 2**		**Isolate 3**		**Isolate 4**	
Amikacin	≤ 4	S	32	R	32	R	16	R
Cefazolin	8	R	8	R	8	R	8	R
Cefepime	≤ 0.5	S	8	SDD	32	R	16	R
Ceftriaxone	≤ 1	S	≤ 1	S	≤ 1	S	≤ 1	S
Ceftazidime	≤ 0.5	S	≤ 0.5	S	≤ 0.5	S	≤ 0.5	S
Ciprofloxacin	> 4	R	> 4	R	4	R	> 4	R
Ertapenem	≤ 0.25	S	≤ 0.25	S	≤ 0.25	S	≤ 0.25	S
Gentamicin	≤ 1	S	4	I	2	S	≤ 1	S
Levofloxacin	> 8	R	4	R	4	R	4	R
Pip/Tazo	≤ 8	S	> 128	R	128	R	> 128	R
Tobramycin	2	S	> 16	R	> 16	R	> 16	R
Trimeth/Sulfa	> 4/80	R	> 4/80	R	> 4/80	R	> 4/80	R

Minimal inhibitory concentration (MIC) is measured in mcg/mL. Resistant, R; Susceptible, S; SDD, susceptible dose dependent. Patient 1, Isolate 1 (UCLA 2066): toe bone collected 1/5/25; Isolate 2 (UCLA 2046): foot bone collected 2/9/25; Patient 1, Isolate 3 (UCLA 2047): blood collected 2/7/25; Patient 2, Isolate 4 (UCLA 2288): hip wound collected 9/9/25.

Over the next two weeks he underwent outpatient revascularization with right-sided superficial femoral and anterior tibial artery angioplasty. Despite this, he had worsening necrosis of the right hallux requiring outpatient disarticulation and amputation to the level of the 1st metatarsophalangeal joint. He returned to the hospital one month after discharge with fevers, chills, nausea, and foul-smelling discharge again from the right foot with concern for treatment failure. He was found to have a white blood cell count of 9.67k/μL, ESR 85 mm/hr, CRP 18.3 mg/dL, and procalcitonin 4.59 ug/L. A follow-up MRI showed progressive osteomyelitis of the remaining first metatarsal shaft and head of the second metatarsal and proximal phalanx with marrow edema in the third metatarsal head and proximal phalanx. He was empirically switched to meropenem and underwent surgical resection of the distal 1st metatarsal bone and partial 2nd ray amputation. Two sets of blood cultures from admission and proximal bone cultures all grew *P. mirabilis*. The blood isolates remained highly susceptible to ceftriaxone (MIC < 1 mcg/mL) but were resistant to cefepime (MIC 32 mcg/mL) (Table 1, Isolates 2 and 3). He was continued on meropenem for a total of 10 days and was later discharged on ertapenem for a planned 6-week course. He was seen in clinic 5 weeks after discharge and was doing well without signs or symptoms of infection. However, he developed recurrent infection with cefepime-susceptible *P. mirabilis* a month later, likely due to inadequate source control requiring further debridement.

Patient 2 is a 68-year-old male with a history of diabetes mellitus type 2, hypertension and hyperlipidemia, who sustained multiple injuries during a motor vehicle accident including complex fractures of the pelvis and left acetabulum in 2023. He had a prolonged ICU course requiring several surgeries including pelvic fracture repair with hardware placement and open reduction internal fixation (ORIF) of the left hip. His post-ORIF course was complicated by infection, and he underwent incision and drainage of the left hip prosthesis and intraoperative cultures grew *P. mirabilis*. The patient was then treated with intravenous ertapenem 1 g daily for 1 month. Over the next year from 2024 to 2025, he continued to have drainage of fluid from the left hip which continued to grow *P. mirabilis* due to hardware infection. As it was deemed too high risk to remove the implanted hardware, he was placed on chronic suppressive cephalexin 500 mg orally 4-times a day instead.

Six months after starting cephalexin, with good adherence to taking the antibiotic, the wound reopened. He underwent another incision and drainage of the left hip and intraoperative cultures grew multi-drug resistant *P. mirabilis* that was resistant to cephalexin. He was taken off cephalexin and placed on cefpodoxime 200 mg orally twice a day instead for chronic suppression. Susceptibility testing confirmed sensitivity to cefpodoxime. Four months later he was found to have breakthrough infection in the pelvis despite being on suppressive cefpodoxime and underwent another debridement. Cultures from the pelvis again grew MDR *P. mirabilis* which was still sensitive to cefpodoxime. A decision was made to completely remove the hardware as there were limited oral antimicrobial options available to suppress the infection. Culture of the hardware grew MDR *P. mirabilis* and the patient completed another 8-week course of intravenous ertapenem.

## Results

Phylogenetic analysis of isolates 1, 2, and 3 from patient 1 demonstrated zero SNPs indicating that they are the same strains (data not shown). Isolate 4 from patient 2 differ from patient 1 isolates by 23 SNPs, and there was no epidemiological link between the 2 cases. The isolates contained genes predicted to confer resistance to several classes of antibiotics including aminoglycosides (*aadA2*, *aph(3')-Ia*, *aph(6)-Id*, *aph(3′')-Ib*), macrolides (*ere(A)*), fluoroquinolones (*aac(6′)-Ib-cr*), phenicol (*catB3*, *floR*, *cat*), sulphonamide (*sul2*, *sul1*), tetracyclines (*tet(A)*, *tet(C)*, *tet(J)*), rifampicin (*ARR−3*), trimethoprim (*dfrA32*), and beta-lactam (*bla*_OXA−1_) (Table 2).

**Table 2:** AMR gene and copy number by WGS analysis, rounded to the nearest whole number.

Gene Category	**Gene**	Isolate 1	Isolate 2	Isolate 3	Isolate 4
**Aminoglycoside**	aph(6)-Id	1X	1X	1X	1X
	aph(3')-Ia	1X	2X	6X	1X
	aadA2	1X	3X	1X	1X
	aph(3′')-Ib	1X	1X	1X	1X
**Beta-lactam**	blaOXA−1*	1X	26X	18X	6X
**Fluoroquinolone & aminoglycoside**	aac(6′)-Ib-cr*	1X	26X	18X	6X
**Macrolide**	ere(A)	1X	1X	1X	1X
**Phenicol**	catB3*	1X	26X	18X	6X
	flor	1X	1X	1X	1X
	cat(A)	1X	1X	1X	1X
**Rifampicin**	ARR−3*	1X	26X	18X	6X
**Sulphonamide**	sul1*	1X	26X	18X	6X
	sul2	1X	1X	1X	1X
**Tetracycline**	tet(A), tet(C), tet(J)	1X	1X	1X	1X
**Trimethoprim**	dfrA32	1X	1X	1X	1X

* AMR genes encoded in the same IS*26* family transposon.

**Fig. 1:**
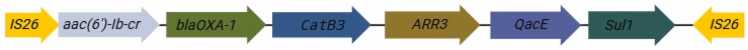
Structure of IS*26* family transposon ∼6 kb. IS*26*: insersion sequence 26 transposase enzyme; *aac(6′)-Ib-cr*: an aminoglycoside acetyltransferase; *bla*_OXA−1_: class D β-lactamase; *catB3*: chloramphenicol O-acetyltransferase; *arr−3*: rifampin ADP-ribosyl transferase; *qacEΔ1*: small multidrug resistant efflux transporter conferring res istance to quaternary ammonium compounds; *sul1*: sulfonamide resistance gene. (Created in https://BioRender.com).

Polished hybrid assembly revealed a resistance cassette containing *aac(6′)-Ib*, an aminoglycoside acetyltransferase associated with quinolone resistance; *bla*_OXA−1_, a class D β-lactamase; *catB3*, a chloramphenicol O-acetyltransferase; *arr−3*, a rifampin ADP-ribosyl transferase; *qacEΔ1*, a small multidrug resistant efflux transporter conferring resistance to quaternary ammonium compounds, and *sul1* which confers resistance to sulfonamide antibiotics (Fig. 1).

**Fig. 2:**
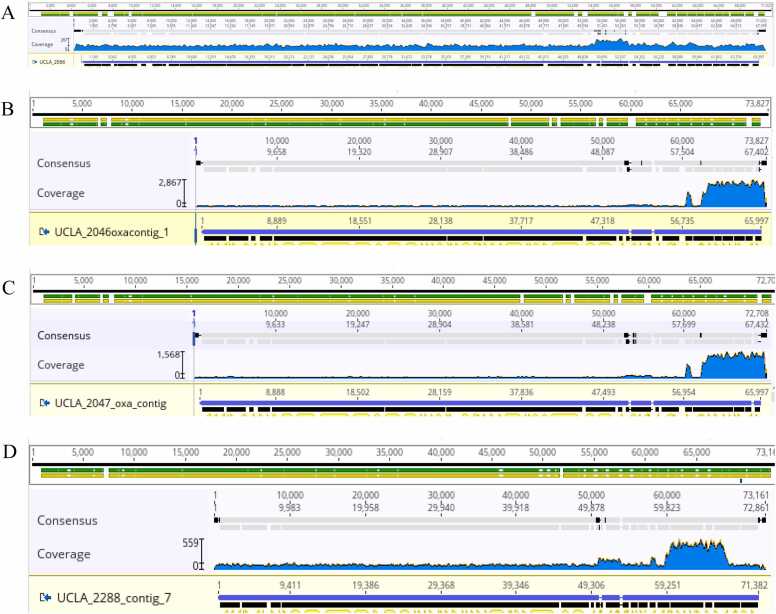
**Gene mapping analysis showing gene duplication.** The short Illumina raw reads from each isolate were mapped back to the OXA-containing contig assebled by long-read Nanopore sequencing. Panel A shows read depth of the cefepime susceptible isolate (UCLA_2066) was even across the entire OXA contig, consistent with no gene duplication. Panel B - D showed reads from the cefepime resistant isolates mapped to the OXA contig, demonstrating markedly increased read depth in just the OXA cassette region, consistent with gene duplication.

Annotation and BLAST analysis against an insertion sequence database identified a flanking transposase belonging to the IS6 family (IS26-like), a class of mobile elements known to mediate replicative transposition and gene amplification [16]. Across all three resistant isolates, the *bla*_OXA−1_ locus demonstrated consistent enrichment relative to chromosomal background (Fig. 2), with asymmetric flanking coverage (Supplemental Figure S1), indicating duplication of a discrete resistance cassette rather than isolated gene amplification or plasmid acquisition; 15–20-fold increase in *bla*_OXA−1_ gene copy number for patient 1 and ∼6-fold increase for patient 2 (Supplemental Table S1). Given the IS6-family activity and the presence of the transposase sequences flanking the cassette, these findings are consistent with IS-mediated duplication of the resistance region. The repetitive nature of this region along with the observed gradated read depth are consistent with IS-mediated duplication and collapse of repeated cassette structures in assembly (Fig. 2; Supplemental Figure S1).

Although *P. mirabilis* is known to be resistant to beta-lactams through plasmid-mediated cephalosporinases, our hybrid assembly does not support a multicopy plasmid carrying *bla*_OXA−1_; coverage is more consistent with a collapsed multicopy transposon. Using NCBI BLAST, the transposon was found to be present in the chromosomes of numerous *P. mirabilis* strains. It was also found to be present in plasmids in *Escherichia coli* and *Salmonella enterica*, and the chromosomes of *Klebsiella pneumoniae* and *Vibrio cholerae*. The cassette-containing contig showed similarity to previously described class 1 integron-associated resistance regions, including class 1 integron In37-like (AY259086) structures previously seen in *K. pneumoniae*, *E. coli*, *S. enterica*, *P. mirabilis* and In*2054*-like (CP068445) structures in *Citrobacter freundeii* [17], [18]. Sequence alignment showed the internal sequence containing all the 6 AMR genes inside the IS26 transposon is nearly identical with the first half of the internal sequence in In*37* (AY259086) and the entire internal sequence of In*2054* (CP068445) (Supplemental Figure S2).

Since *bla*_OXA−1_ was the only beta-lactamase gene found in these isolates, we further investigated its role in causing the unusual resistance pattern. Compared to isolate 1, isolates 2 and 3 had the gene coverage depth increased by 18x and 26x, respectively (Table 2). Isolate 4 has a relative *bla*_OXA−1_ gene coverage depth of 6x. The other genes associated with the same degree of gene duplication included *aac(6′)-Ib-cr, ARR−3, catB3,* and *sul1*, all from the same IS*26* family transposon (Fig. 1), suggesting the gene duplication was occurring at the transposon level. The use of short-read–polished assemblies and uniform contig coverage supports true biological amplification rather than assembly artifact or mapping bias. As a control, the *tet(C)* gene which was not under selective pressure and not found in the same transposon and remained a single copy in all isolates. Genes encoding porin proteins in *P. mirabilis* associated with drug resistance including *omp*A and *opr*D were also analyzed but no porin mutation was detected in any isolates.

## Discussion

*Proteus mirabilis* is a commensal organism of the human gastrointestinal tract that is often isolated from various environmental sources such as soil and sewage water. A frequent cause of urinary tract infections, it can also cause wounds, ocular, pulmonary and bloodstream infections [19]. Especially in the outpatient setting, *P. mirabilis* can be challenging to treat due to intrinsic resistance to tetracycline, tigecycline, nitrofurantoin, polymyxins coupled with increased levels of resistance to imipenem due to loss of outer membrane porins, decreased expression of PBP1a or reduced affinity of imipenem for PBP2 [20], [21]. Treatment options for *P. mirabilis* usually includes aminopenicillins, second- and third-generation cephalosporins, trimethoprim–sulfamethoxazole, and fluoroquinolones [22].

Although not common, cefepime-resistant *P. mirabilis* has been reported in the United States and other countries. SENTRY data from 2009 to 2011 found less than 1% of *P. mirabilis* isolates collected from ICU and no-ICU patients in the US were cefepime-resistant, and less than 7% of isolates from hospitals in Europe in the same time frame were resistant to cefepime [23]. Cefepime-resistant *P. mirabilis* strains have also been reported from hospitals in countries such as Egypt and Taiwan and most isolates expressed extended spectrum beta-lactamase (ESBL) genes [24]. A recent study in India analyzed 100 *P. mirabilis* clinical isolates; 33.3% of isolates were resistant to commonly used antibiotics with the highest resistance noted against ciprofloxacin (58%), ceftazidime (50%), and cotrimoxazole (49%) [25]. Additional resistance was noted against ceftriaxone (35%), cefepime (30%), amoxicillin-clavulanate (24%), and meropenem (23%). Of the resistant isolates, 19% carry the blaNDM−1 gene, 8% were positive for blaKPC−2 genes, and 4% of the isolates co-harboring both [25].

There have not been any reported cases of cefepime-resistant and ceftriaxone-susceptible *P. mirabilis* to date. However, this unusual pattern of resistance to cefepime but susceptibility to 3rd generation cephalosporins has been described in *P. aeruginosa* and was attributed to presence of *bla*_OXA−1_ or *bla*_OXA−31_ genes [26]. A similar resistance pattern was also reported in *E. coli* due to the expression of *bla*_OXA−1_ [27]. Both studies reported no deficiency in outer membrane porins or efflux pumps. Torres et al., 2016 characterized the resistance phenotype and molecular epidemiology of 33 Enterobacterales isolates which included 27 *E. coli*, 4 *K. pneumoniae*, 1 *P. mirabilis*, and 1 *Salmonella* spp. that had reduced susceptibility to cefepime and amoxicillin/clavulanate and increased susceptibility to oxyimino-cephalosporins [28]. They reported that this phenotype was mainly due to the presence of *bla*_OXA−1_, particularly when it was combined with other resistance mechanisms such as *bla*_TEM−1_ and porin loss. However, the *P. mirabilis* isolate analyzed in that study only carried *bla*_TEM−1_ and not *bla*_OXA−1._

In our study, *bla*_OXA−1_ was the only beta-lactamase gene in all *P. mirabilis*. There was also no evidence of porin mutations. These results suggest that the gene duplication of *bla*_OXA−1_ is the main resistance mechanism for cefepime and piperacillin/tazobactam in the resistant isolates (2, 3, and 4) that emerged after prolonged antibiotic treatment. Further studies are needed to determine if the resistant phenotypes would revert to more susceptible AMR profile in the absence of selective pressure from the antibiotics. Importantly, the gene duplication of *bla*_OXA−1_ did not seem to affect susceptibility to ceftriaxone. It is not clear why *bla*_OXA−1_ gene duplication only affected cefepime. One possible explanation is the structural difference between 3rd and 4th generation cephalosporins. Fourth generation cephalosporins such as cefepime and cefpirome have a quaternary ammonium group at the C−3′ position which is lacking in the 3rd generation cephalosporins [29]. It has been suggested that OXA−1 and its derivatives may selectively hydrolyze some 2-amino−5-thiazolyl cephalosporins such as cefepime and not ceftazidime or cefotaxime [26].

The mechanism by which *bla*_OXA−1_ duplicates has not been investigated. One of the limitations of this report is that although we used long read sequencing to compare AMR gene coverage depth to chromosomal coverage depth, fold depth increase does not provide exact copy numbers. Hubbard et al., 2020 reported a phenotype of piperacillin-tazobactam resistant but carbapenem and 3rd generation cephalosporin susceptible *E. coli* and *K. pneumoniae* [16]. This was due to IS*26*-mediated hyperproduction of *bla*_TEM−1B_ through a series of excision and reinsertion of translocatable unit (TU). It is reasonable to hypothesize that *bla*_OXA−1_ duplication in *P. mirabilis* in the cases presented in this study may occur through a similar mechanism; however, further investigation is needed.

In conclusion, we demonstrated that gene duplication of a narrow-spectrum penicillinase OXA−1 can be associated with cefepime resistance in *P. mirabilis* while remaining susceptible to 3rd generation cephalosporins such as ceftriaxone. The prolonged antibiotic treatment, including cefepime and ertapenem, was most likely responsible for *bla*_OXA−1_ gene duplication at the transposon level. We also found the IS*26* family transposon carrying *bla*_OXA−1_ along with 5 other AMR genes widely circulating in plasmids or chromosomes of *P. mirabilis* and many other Enterobacterales species. Clinicians should be aware of this unusual resistance mechanism, especially in chronic infections with the potential for treatment failure in the outpatient setting. More frequent monitoring of the antimicrobial susceptibility of the clinical isolates is warranted.

## CRediT authorship contribution statement

**Anita Sircar:** Writing – review & editing, Investigation, Data curation. **Shekina Gonzalez-Ferrer:** Writing – review & editing, Visualization, Investigation, Formal analysis, Data curation. **Mario Jaramillo:** Writing – review & editing, Investigation, Data curation. **Mackenzie Collins:** Writing – original draft, Visualization, Validation, Project administration, Methodology, Investigation, Formal analysis, Data curation. **Amorce Lima:** Writing – original draft, Visualization, Validation, Project administration, Methodology, Investigation, Formal analysis, Data curation. **YANG SHANGXIN:** Writing – review & editing, Validation, Supervision, Software, Resources, Investigation, Funding acquisition, Formal analysis, Conceptualization. **Paul R. Allyn:** Writing – review & editing, Investigation, Data curation.

## Declaration of Competing Interest

All authors declare no conflict of interests.

## Footnotes

Appendix A Supplementary data associated with this article can be found in the online version at doi:10.1016/j.idcr.2026.e02654.
