# Supplementary material for: Cefepime resistance with preserved ceftriaxone susceptibility in Proteus mirabilis osteomyelitis associated with blaOXA-1 gene amplification: Two case reports with genomic analysis
Source: IDCases. 2026 Jun 25;45:e02654. doi: 10.1016/j.idcr.2026.e02654 (PMC13318549; doi:10.1016/j.idcr.2026.e02654)
Supplement: Supplementary file 1 — Supplementary material [file mmc1.docx]

**Supplemental Table S1**. **OXA copy number analysis.**

| **Metric** | **Isolate 1 (Case 1, toe)** | **Isolate 2**  **(Case 1, foot)** | **Isolate 3**  **(Case 1, blood)** | **Isolate 4**  **(Case 2, hip)** |
| --- | --- | --- | --- | --- |
| L flank avg depth | 138.005 | 1332.02 | 753.86 | 233.234 |
| OXA avg depth | 113.377 | 2632.31 | 1443.77 | 443.31 |
| R flank avg depth | 111.67 | 2715.12 | 1497.51 | 451.248 |
| OXA-contig avg depth | 125.886 | 389.77 | 246.225 | 126.083 |
| Chromosome avg depth | 95.0153 | 106.526 | 89.205 | 76.626 |
| OXA/L flank fold | 0.821 | 1.976 | 1.915 | 1.900 |
| OXA/R flank fold | 1.015 | 0.969 | 0.964 | 0.982 |
| OXA/OXA-contig fold | 0.900 | 6.753 | 5.863 | 3.516 |
| OXA/chromosome fold | **1.193** | **24.710** | **16.184** | **5.785** |

Left flank and right flank are 2.5kb up and downstream, depth is Illumina reads mapped to hybrid assembly, OXA-contig is the ~66kb cassette-containing contig.

A


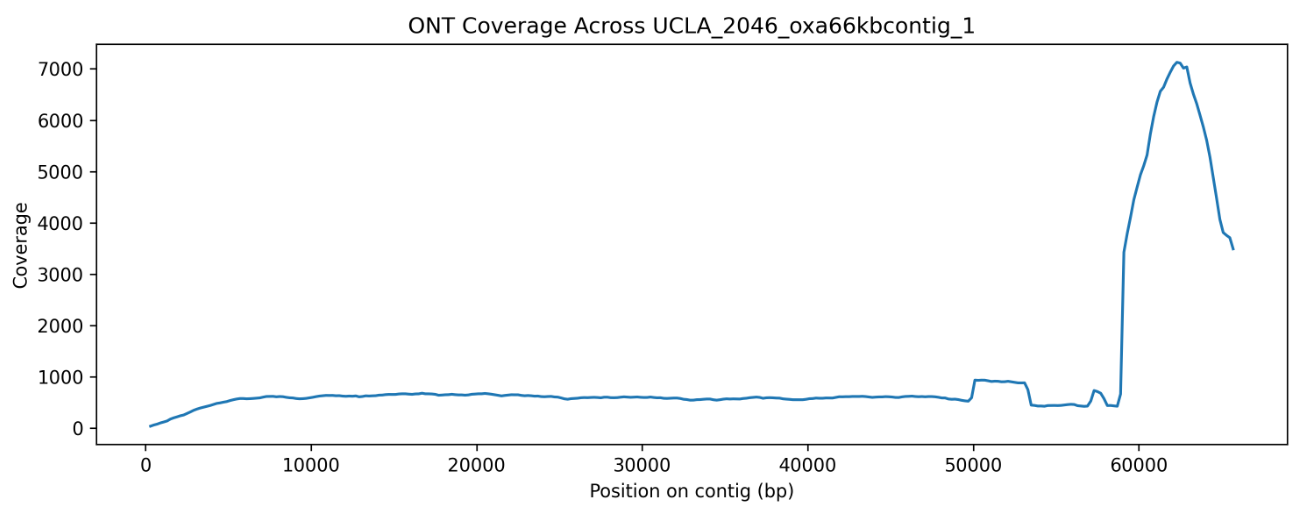


B


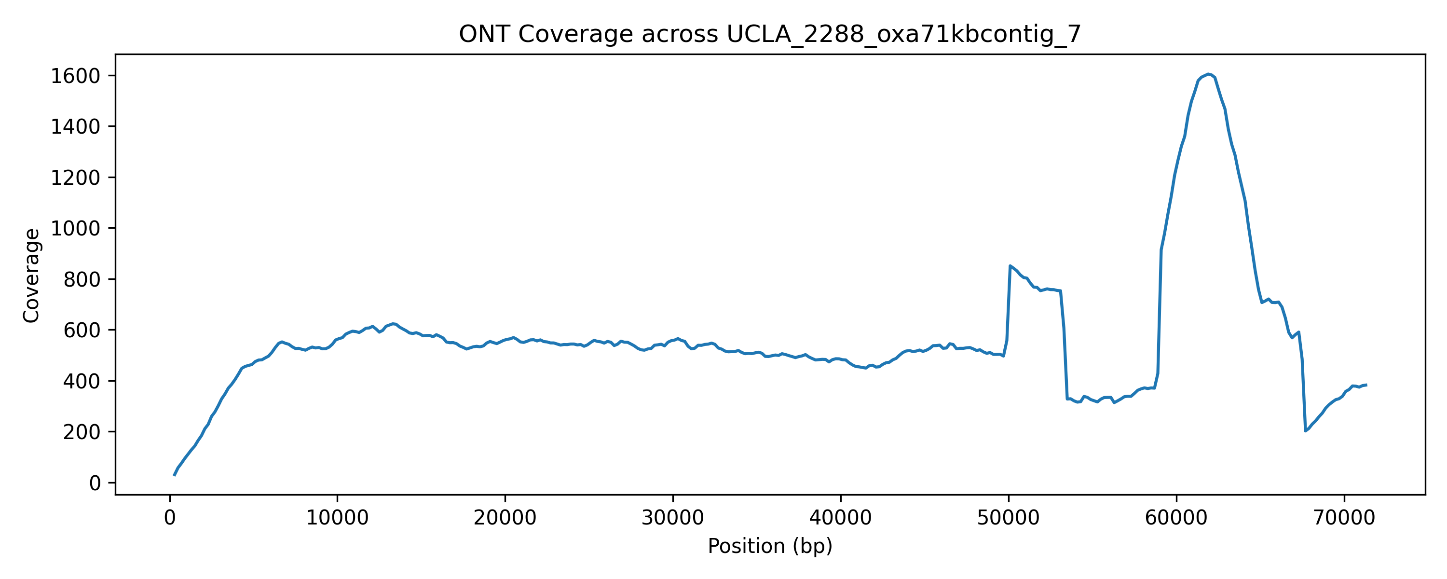


**Supplemental Figure S1.** One resistant isolate from patient 1 (UCLA_2046) and the resistant isolate from patient 2 (UCLA_2288) were examined to estimate OXA gene copy number using just long reads. The graphs show coverage depth with ONT long reads mapped to the OXA-containing contig. Both isolates showed markedly increased coverage over the roughly 6kb cassette within the larger contig, which had baseline chromosomal coverage. This was consistent with the results obtained mapping only short reads to the OXA contig, further confirming gene duplication as the mechanism of resistance.

B


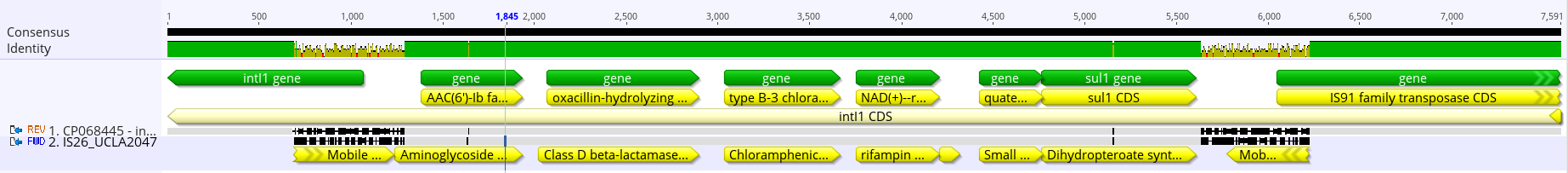


A


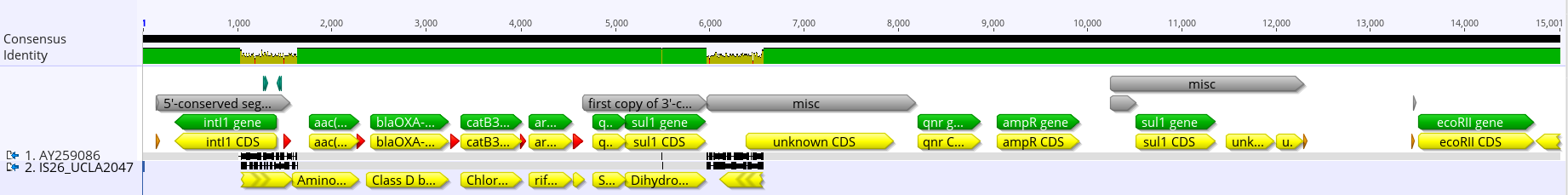


**Supplemental Figure S2.** Comparison of Mobile Genomic Elements carrying OXA-1. A. IS*26* gene cassette *aac(6′)-Ib cr* – *bla*_OXA 1_ – *catB3* – *arr 3* – *qacEΔ1* – *sul1* alignment with In*37* (AY259086). B. IS*26* gene cassette alignment with In*2054* (CP068445).
